# Duty, desire or indifference? A qualitative study of patient decisions about recruitment to an epilepsy treatment trial

**DOI:** 10.1186/1745-6215-7-32

**Published:** 2006-12-12

**Authors:** Krysia Canvin, Ann Jacoby

**Affiliations:** 1 Division of Public Health, University of Liverpool, Liverpool, UK

## Abstract

**Background:**

Epilepsy is a common neurological condition, in which drugs are the mainstay of treatment and drugs trials are commonplace. Understanding why patients might or might not opt to participate in epilepsy drug trials is therefore of some importance, particularly at a time of rapid drug development and testing; and the findings may also have wider applicability. This study examined the role of patient perceptions in the decision-making process about recruitment to an RCT (the SANAD Trial) that compared different antiepileptic drug treatments for the management of new-onset seizures and epilepsy.

**Methods:**

In-depth interviews with 23 patients recruited from four study centres. All interviews were tape-recorded and transcribed; the transcripts were analysed thematically using a qualitative data analysis package.

**Results:**

Of the nineteen informants who agreed to participate in SANAD, none agreed for purely altruistic reasons. The four informants who declined all did so for very specific reasons of self-interest. Informants' perceptions of the nature of the trial, of the drugs subject to trial, and of their own involvement were all highly influential in their decision-making. Informants either perceived the trial as potentially beneficial or unlikely to be harmful, and so agreed to participate; or as potentially harmful or unlikely to be beneficial and so declined to participate.

**Conclusion:**

Most patients applied 'weak altruism', while maintaining self-interest. An emphasis on the safety and equivalence of treatments allowed some patients to be indifferent to the question of involvement. There was evidence that some participants were subject to 'therapeutic misconceptions'. The findings highlight the individual nature of trials but nonetheless raise some generic issues in relation to their design and conduct.

## Background

Within the framework of biomedicine, randomised controlled trials (RCTs) are seen as providing the most valid means of establishing the efficacy of treatment for conditions of ill health but are inherently difficult to recruit to 'precisely because of the randomisation element that makes them so statistically attractive' [1]. Low recruitment impacts on both the progress and scientific quality of trials [2,3], with many either failing to start or stopping due to lack of participation, and those that continue being subject to potential bias because of failure to recruit representative samples of patients. This has stimulated concern among trialists about how to maximise recruitment rates and consequently, interest in the processes around patients' decision-making concerning participation. Previous studies have explored both generalised attitudes towards hypothetical RCTs among patients and the general public [4-9] and the perceptions of real-life trial participants about their experience of participation [10-13]; have involved both healthy and non-healthy trial participants and their proxies [14-17] and potentially eligible patients [18-21]; have encompassed similar and highly contrasting treatments [14-16,18-20,22] and have been conducted across varying states of health emergency and non-emergency [16,17,21,24]. These studies have highlighted both drivers and barriers to patient participation.

Large among suggested drivers for participation in clinical trials is the notion of altruism [6-14,24]. Indeed, Emmanuel and Patterson [25] go so far as to state that 'the only legitimate justification' for randomisation is 'that the patient chooses to be altruistic.' Certainly the idea that patients should be involved in research is strongly endorsed by non-patients and patients alike [5,8-11,14]. In the study by Searight & Miller [14], patients often emphasised taking part in clinical trials as performing a valuable service to the community of sufferers to which they belonged. However, studies exploring motivations for taking part in medical research, including trials, have found that patients are also driven by reasons of self-interest, seeing it as a means of ensuring treatment by a specialist or securing the best available therapies and medical care [6-13,15,16,26]. Schutta and Burnett [18] note that in their investigation, patients expressed surprise that people would participate in clinical trials for altruistic reasons. Both individual studies [6-9,14] and systematic reviews of the literature [2,3,27] have so far drawn opposing conclusions about the relative weight given to altruism and self-interest.

Other factors highlighted as motivating patients to participate in clinical trials include the trust they hold in their doctor [22,23] and in the study [20]. Less positively, patients may be influenced to take part because they hold 'therapeutic misconceptions' [28] about what involvement will mean. Lidz et al [28] have argued that therapeutic misconceptions arise from patients' failure to 'grasp that the risks they face from participating in research protocols are inherently different from those involved in receiving ordinary treatment' and when they inaccurately attribute, 'a primacy of therapeutic intent and individualized care typically seen in ordinary clinical settings to research procedures.' These authors highlight a number of studies in addition to their own suggesting that therapeutic misconception on the part of trial participants is not uncommon, with subjects underestimating risks [29], over-estimating benefits [30] and conflating research and ordinary treatment [29].

Potential barriers to participation in clinical trials have been identified as: unwillingness to accept the possibility of clinical uncertainty and equipoise [4,20,25]; unwillingness to accept the idea of treatment allocation at random [4-6,17,19,23], the possibility of being randomised to placebo rather than active treatment [13,21,23] and holding clear preferences for a particular method of management [26]; worry about receiving 'experimental' treatments [6] or being used as 'guinea pigs' [8,24]; reluctance to change treatment [7]; the belief that trial participants might be denied access to new treatments or that the trial did not offer the best available treatment [21,23] and the view that any benefits of taking part would not outweigh the risks [15,21,24,26]. Distrust of the medical profession and lack of knowledge of what is required of trial participants have also been highlighted as potential barriers [22]; as has inconvenience and discomfort [2,12] and fear of side-effects [7,26]. Fallowfield at al [5] identified three distinct groups of patients with regard to clinical trial participation: those for whom the concept of randomisation was acceptable and who would therefore agree to participate in clinical trials; those who had concerns about the concept, but were nonetheless open to considering participation; and those who were firmly against randomisation and participation in trials.

While patient-related factors are clearly crucial to the issue of trial recruitment, investigators have also identified other clinician- and trial-related factors that may influence recruitment rates. Systematic reviews have [2,3,22] listed among clinician-related factors logistic difficulties such as lack of time and resources, and difficulties with ethical requirements; and personal difficulties such as discomfort with randomisation and preference for a particular treatment, concerns about the effect on the doctor-patient relationship, and difficulty with informed consent procedures. Roberts [31] and Donovan et al [32] have both explored qualitatively how different clinicians' presentation of the same clinical trial can vary substantially, with important implications for recruitment. Trial factors include poorly designed or complex protocols, presence of a no-treatment arm or too large a difference between active treatments, and concerns about treatment toxicity [22].

Based on their review of the existing literature, Ross et al [3] conclude that further research is needed to understand more clearly reasons why patients do or do not take part in clinical trials; and that such work can be nested within ongoing trials. We have examined the issue of participation in clinical trials in the context of a common chronic condition, epilepsy, and a non-controversial intervention, in which six different antiepileptic drugs (AEDs) were compared [33]. Epilepsy is the commonest serious brain disorder [34]; and is characterised by recurrent unprovoked seizures which in most patients can be successfully treated and controlled [35]. Though there is an expanding surgical programme for epilepsy worldwide, drugs remain the mainstay of treatment. Recent advances in understanding of the basic mechanisms for seizures have led to a period of rapid drug development and testing, so that drug trials are now commonplace. Understanding why patients might or might not opt to participate in epilepsy drug trials is therefore of some importance within the field itself, but may also offer some more general insights. Surprisingly, given the level of activity in the field, we are unaware of any of studies exploring this issue in relation to epilepsy trials.

Studies exploring patients' reasons for participation or non-participation have so far focussed on trials that have involved testing new or markedly different treatments [20,32] and trials conducted in situations of clinical emergency [17,23] or of treatment for life-threatening conditions [5,10,18], which have therefore proved difficult to recruit to. The situation around which our study was framed was in marked contrast to these, in that the UK SANAD study was a trial of six currently available drug treatments for a chronic and, in the main, clinically benign condition, epilepsy. (SANAD is the acronym for '**S**tandard **A**nd **N**ew **A**ntiepileptic **D**rugs'; SANAD was funded by the UK NHS Health Technology Assessment Programme; ISRCTN number 38354748.)Patients potentially eligible for SANAD were those with a newly confirmed diagnosis of epilepsy, for whom treatment with a single AED (monotherapy treatment) was indicated. SANAD proved a highly successful trial, recruiting over 2,000 such individuals, from 90 centres across the UK. It therefore presented an interesting and timely opportunity to explore, using a nested study design, the role of patient perceptions and understandings in the decision-making process about recruitment to epilepsy drug trials; and their *actual *rather than hypothetical reasons for either agreeing or declining to participate.

Unusually in the context of clinical trials, all the drugs included in SANAD were already available to patients within the NHS, though two were not licensed for use as monotherapy at the time of trial recruitment. The two standard drugs, Sodium Valproate and Carbamazepine, are currently considered to be first-line treatments for epilepsy, with the other four recently licenced drugs (Lamotrigine, Oxcarbazepine, Gapapentin, Topiramate) being prescribed, in the main, only when they have failed. In spite of this, there has been a steady rise in the prescribing of the newer AEDs from 0.1% of total AED prescriptions in 1991 to 20% in 2002. Because of the much higher costs of these newer drugs relative to the standard ones, a recent NICE appraisal estimated that they now account for 69% of the total costs of AEDs to the NHS [36]. SANAD was therefore funded as a Phase IV trial to provide information on the clinical utility of these newer AEDs. The aims of our nested 'participation' study were:

1. to examine adult patients' and parents reasons for agreeing or declining to take part in the SANAD trial;

2. to explore their perceptions of SANAD and understanding of the nature of this particular clinical trial, and how these perceptions and understandings contributed to their enrolment decisions;

3. to consider whether there were lessons to be learned about how the recruitment process in future trials in epilepsy might be improved.

## Methods

As outlined above, SANAD was a multi-centre, pragmatic, unblinded, parallel-group RCT, which compared monotherapy with clinicians' first-choice standard AED versus appropriate comparators from among the newer AEDs [33]. By the end of the 6-year recruitment phase in August 2004, almost two and a half thousand individuals aged over five years (92% of all eligible individuals) had been randomised to SANAD. The majority of those randomised were adults (81%, see Table 1); and a somewhat higher proportion of decliners were parents of children aged under 16 years. In all, 6% of eligible adults and 11% of parents of eligible children declined to take part.

**Table 1:** Recruitment to SANAD Trial

	**Adults**	**Children**	**All**
Eligible	2119 (81%)	508 (19%)	2627 (100%)
Randomised	1987 (82%)	450 (18%)	2437 (100%)
Declined randomisation	132 (69%)	58 (31%)	190 (100%)

Recruitment to SANAD took place within a consultation at which the clinician first had to confirm a diagnosis of epilepsy. Typically, recruitment consultations began with a detailed history taking and examination, at the end of which the clinician reached a conclusion about whether or not the patient was experiencing seizures. If a diagnosis of epilepsy was confirmed, the clinician then discussed its implications, both in relation to broader 'quality of life' issues and to treatment options specifically, including the likelihood of a seizure recurrence if treatment was withheld. For the patient, confronting the decision whether to begin treatment and uncertainties about treatment outcomes was thus a major element of the consultation. Only when patients accepted the need for treatment were they approached to take part in SANAD. They were given oral and written descriptions of the trial, which explained its rationale, the process of randomisation, possible risks and benefits of participation, the length of the trial (six years), and detailed information about the trial drugs and their potential side effects. The trial information sheet highlighted that, drugs used to treat seizures were 'generally safe' and that the study '*may *benefit you personally and it *will *also benefit other people who have seizures and epilepsy.' Most patients made their decision about participation in SANAD on the same day as they were approached.

In order to recruit to the present 'participation' study, one of the authors (KC), who was not otherwise involved in the SANAD trial, attended 37 clinics (approximately 48 clinic hours) over a 13-month period across four hospitals. The decision to limit recruitment to only four centres (two adult and two paediatric neurology clinics at three hospitals (A,B,C) in North West England and one in North Wales (D)) was made for practical and logistic reasons, all being easily accessible to the researchers. When contact was made at clinic with eligible patients, KC gave them printed information about the 'participation' study and asked if they would be willing to be interviewed for it, at a time and venue suitable for them.

The number of new patients (i.e. potentially eligible for the SANAD trial and, in turn, for the participation study) was approximately 8 per clinic. Therefore, the researchers potentially had access to a pool of approximately 315 potential participants. We aimed to recruit up to 20 adults and up to 20 parents who were making the decision about recruitment to SANAD on behalf of their child, at the point of randomisation; to include both consenters and decliners in each group; and to interview them within a month of the recruitment consultation. In the event, our recruitment strategy proved less successful than we hoped, although just five patients refused to take part in the 'participation' study. Considerably fewer patients were approached (a total of 43) for a number of reasons. First, the pool of potential patients at each clinic attended was considerably less than expected because patients proved to be ineligible for SANAD (approximately 68% of those not approached). For example, some were discharged without a positive diagnosis and referred for further tests or follow up. Second, some eligible patients failed to attend the clinic appointment (approximately 24%). Third, the logistics of clinic organisation meant some were not flagged as eligible to KC (approximately 8%).

Furthermore, a high initial consent rate to the 'participation' study (88%, 38 of 43 eligible individuals who consented to SANAD) fell to 51% (23/43) because 15 patients agreeing to be interviewed when initially approached either could not be re-contacted for an interview appointment, cancelled or failed to keep one that had been agreed, or subsequently proved to be ineligible for SANAD (Table 2). Of the 15, one had declined participation in SANAD, one withdrew after consenting initially and the remaining 13 had consented. This report is therefore based on 23 individuals interviewed, of whom 15 (65%) were adult informants and eight were parents (Table 3). Four patients (17%), all adults, had declined to participate in SANAD and one, also an adult, had agreed initially and then withdrawn. Of the 2,437 individuals consenting to randomisation to SANAD, only 47 (2%) subsequently withdrew consent. None of the parents we interviewed had declined to allow their child to be entered into SANAD, though one had decided not to take part initially and was then persuaded by her discussion with the clinician to reverse her decision.

**Table 2:** Reasons for informants recruited but not interviewed

**Number of informants recruited but not interviewed**	**Reason**
2	Interviewer unable to re-contact to make appointment
3	Patient cancelled appointment
4	Patient did not attend interview appointment
6	Patient proved ineligible for SANAD
15	TOTAL

**Table 3:** Characteristics of informants

**Informants (n = 23)**	**Female/male**	**Adult/parent of child**	**Decision**	**Hospital**
**1**	Female	Parent	Consented	A
**2**	Female	Parent	Consented	C
**3**	Female	Parent	Consented	C
**4**	Female	Parent	Consented	C
**6**	Male	Adult	Consented	B
**7**	Female	Adult	Consented	D
**8**	Male	Adult	Consented	B
**9**	Female	Adult	Consented	B
**10**	Male	Adult	Consented	B
**11**	Female	Adult	Consented	B
**21**	Female	Parent	Consented	A
**22**	Male	Parent	Consented	A
**23**	Female	Parent	Consented	A
**13**	Male	Adult	Consented	D
**15**	Male	Adult	Consented	B
**16**	Male	Adult	Consented	B
**17**	Female	Adult	Consented	B
**19**	Female	Parent	Consented	A
**14**	Male	Adult	Withdrew	D
**18**	Female	Adult	Declined	B
**5**	Male	Adult	Declined	B
**12**	Male	Adult	Declined	B
**20**	Male	Adult	Declined	B

**TOTAL – n (%)**	**Female – 12 (52%)** **Male – 11 (48%)**	**Adults – 15 (65%)** **Parents – 8 (35%)**	**Consented – 18 (78%)** **Declined – 4 (18%)** **Withdrew – 1 (4%)**	**A – 5 (22%)** **B – 12 (52%)** **C – 3 (13%)** **D – 3 (13%)**

Face-to-face, topic-guided interviews were conducted by KC in informants' homes on average 15 days after the invitation to participate in SANAD (range of 3 to 32 days); although in one unusual case, 73 days had elapsed. Informants were first asked to recall the content of the consultation where they had been asked to participate in SANAD, then to describe their feelings, perceptions and understandings about their diagnosis and treatment, and the invitation to participate in SANAD, including the information provided. Informants were asked about the reasons for their participation decision, and prompted to consider the utility of the information received and what they understood about the purpose of SANAD, the issue of clinical uncertainty and the concept of random allocation.

All interviews were tape-recorded, with consent; and transcribed verbatim. The data were analysed using a computerised qualitative data analysis package (Atlas.ti). KC generated codes through open coding and categorised these thematically; relationships between themes were then identified through constant comparison of the transcripts, codes and categories [37]. AJ reviewed the codes and their application and suggested alternative interpretations until consensus was reached about the interpretation that best fitted the data. Although we draw attention here to themes that were more strongly associated with particular groups (e.g. parents or decliners), many themes were cross-cutting.

All quotes in the text have been anonymised: AA denotes an adult patient who agreed to randomisation; PA denotes a parent who agreed to randomisation; AD refers to an adult patient who declined randomisation and PD a parent who declined on behalf of their child.

## Results

### The context of recruitment

As described above, patients were recruited to SANAD during a clinic consultation at which they were first confirmed as having epilepsy. Not surprisingly, even among those who had realised they were experiencing seizures of some kind and had suspected this to be the case, confirmation of their condition was generally unwelcome and for some, something of a shock. An adult patient who, in light of the family history (a close relative with epilepsy), already suspected his symptoms were epilepsy noted that, 'in the back of my mind, I thought they [the symptoms] are very similar, but I really didn't want to have the same' (AA7). Another adult who also believed epilepsy was a hereditary condition described receiving the diagnosis as 'traumatic' because:

'There is no history or trace of history of epilepsy anywhere at all in the family, not just in living memory, but we have spoken about relatives and so on and there has never been epilepsy in the family, so to suddenly be told you're the first one to suffer from this problem, and to have to come to terms with that.' (AA9)

Some informants expressed reluctance to accept the diagnosis; though for others it came as a relief to be able finally to put a label to the symptoms they or their child had been experiencing. However, the element of unexpectedness to the diagnosis meant patients and parents were often unprepared for deciding whether to begin what might prove to be long term drug treatment. It was in this context that their reasons for agreeing or declining to take part in SANAD were made.

### Reasons for agreeing or declining to participate in SANAD

**Figure 1:**
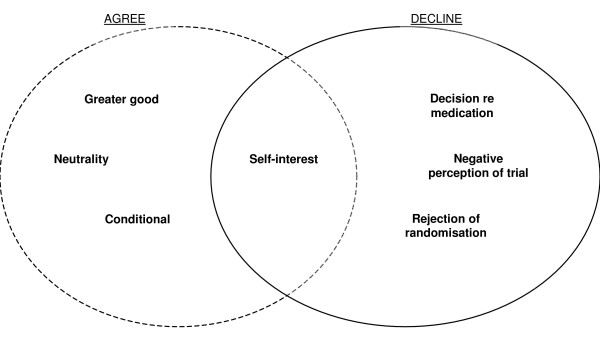
Reasons for agreeing or declining to participate SANAD.

Reasons for participating in the SANAD trial can broadly be seen as falling into one of three categories, which we have defined as a sense of altruism or duty, personal desire and self-interest, and a degree of neutrality or indifference (Figure 1). These categories and the ways in which they overlapped in patients' deliberations, are considered below.

#### Duty as a driver for participation

In keeping with previous studies, all but five participants in SANAD offered altruistic reasons and a sense of moral duty for agreeing to participate, including: the wish to help people with epilepsy; the wish to do their 'bit' and 'give something back' or help the researchers; the perception that they had a duty to participate; and the view that the trial was a 'good idea' or necessary and would contribute to scientific knowledge. However, none of the informants in this study agreed to participate for purely altruistic reasons: all who gave reasons that fell under what can be broadly described as 'the wish to act for the greater good' also gave reasons that represented self-interest and personal 'desire'. Three parent informants clearly articulated their ideas relating to altruism – that trials have to be conducted, and there is a duty to take part in them:

'Because it's got to be proved what, whether or not [...] if people hadn't done this with Epilim and that, then we wouldn't know if people were going to be able to take it or not, it's got to be done.' (PA2)

'I mean it's not advancing medical research if there is people not prepared to go in for the trial, and I think to further, to help medical science, [...] I did it.' (PA19)

'When you think about it every drug they bring out has to be tested in some way or other, hasn't it. So if nobody got tested then we would all be in a right state, wouldn't it. You can only take testing so far in a laboratory can't you?' (PA22)

Yet, these parents also made separate comments indicating that they were also acting in what they saw as their child's best interests and in the hope that she/he would benefit directly.

#### Desire as a driver for participation

All 19 informants who agreed to participate in SANAD, whether or not they proffered altruistic reasons, expressed reasons of self-interest for doing so, including personal preferences and desires about treatment. Some patients actively wanted treatment, rather than no treatment, in order to 'get it sorted out straight away' (AA10) and 'stop this happening' (AA11); and others assumed that participation would lead to *better *treatment. Three informants, all of whom actively wanted treatment, only gave reasons related to self-interest. Several informants perceived taking part in SANAD as having immediate potential benefit for them or their child. Others reasoned that as part of the broader community of people with epilepsy, taking part in SANAD might be of benefit to them in the longer term. The perception of personal benefit, however indirect, was apparent in many informants' responses, even when they also gave altruistic reasons for participating:

KC: 'So why did you decide to take part?'

Informant: 'To help me.'

KC: 'To help you?'

Informant: 'To help me yeah. (...) if it helps other people while they are sorting me out, then well and good.' (AA17)

'Yeah, well, I thought if it helps other people it could help me. 'Cause if I'm helping in it, they might find something else that's linked to it in some way that they didn't know before and then they can go, oh well, yeah that bit matches that, so we can do something about that. So they might be able to work something else out. So that's the idea I've got.' (AA8)

However, perceptions of benefit appeared sometimes to rest on a 'therapeutic misconception' [28]. First, some informants appeared to be attributing 'primacy of therapeutic intent' [28] in as much as they believed that despite the process of randomisation treatment would nonetheless be individualised. Second, some informants appeared to over-estimate the potential benefits of taking part in SANAD, seeing it as the means of securing the 'best' treatment for them or their child:

' [*Consultant*] said he put all the information he had about me, he would put it all in the computer, and then the computer would choose what the computer thought was the best drug for me.' (AA17)

'I'm not a stupid person, I already thought I was saying no, but then like I say, when [*consultant*] explained to me that they were quite safe. I just thought, well, am I denying my child the best treatment – and I thought it is not right for me to be doing this.' (PA19)

#### Indifference to participation

A number of informants – mainly adult patients – appeared to agree to participate less because they could identify positive benefits than because they could not identify any active harms. Taken together, their comments conveyed a sense of neutrality, even indifference, about participation, although the origins of this indifference seemed to vary. For some patients, it appeared predicated on the fact that they had certain expectations for their treatment and once those expectations were fulfilled, they were indifferent to participating in SANAD:

'I knew he was going to get medication anyway, so as long as he's getting medication that's fine. If other people are, you know, using that to see what to do, then that's fine.' (PA4)

Other patients made comments suggesting their indifference was tempered by a weak sense of duty:

' [*Consultant*] mentioned the fact that this study was going on and had been going on for some time, and was I interested in joining in. I said why not, it all had to be done and I was quite happy to go along with [*it*].' (AA16)

'So I thought if I can do my bit, well why not? It's no skin off my nose is it?' (AA8)

Furthermore, a number of patients explicitly referred to aspects of the trial design which may have allowed them to act with apparent indifference. For example, a parent (PA2) thought her child 'might as well be involved' but then said that, had the drugs been experimental, she would have 'been a bit more dubious' about consenting. Finally, one patient's apparent indifference clearly reflected an expression of trust in his doctor:

KC: 'So was it a concern then, that in fact it wasn't the doctor picking the drug, that it was being picked randomly by the computer?'

Informant: 'Not really, because I just thought that the computer is only there, just picking numbers and whatever my number is it is, and whoever programmed it is going to be a doctor, so he is going to know what he is doing – so you have just got to put your trust in the doctor who has done the programme to allocate you to whatever tablets. It doesn't really bother me that much, to be honest.' (AA8)

In contrast, other informants emphasised how they were *not *simply 'going along with' it. Indeed, some – mainly parents – agreed to participate in the trial only on condition that certain beneficial criteria *pertaining to the trial *were fulfilled – in particular that participation would not cause any harm:

'I wasn't going to be pressurised into anything. I am not the type of person that would go along with something I wouldn't do that, I asked a lot of questions before I made my decision and I thought it was the right thing to do.' (PA19)

'As long as it wasn't putting him [*child*] in danger then yes, fine.' (PA4)

#### Desire as a barrier to participation

Informants who declined to participate in SANAD expressed specific desires as to how they wished to proceed following their diagnosis, based on explicit self-interested reasons. These desires acted as a barrier to their participation in SANAD. For example, one informant (AD18) was reluctant to risk randomisation to drug that could interact with the contraceptive pill or, indeed, to begin any treatment at all until she had received counselling:

'Some of the drugs on the trial [*nurse specialist*] said didn't interact well with contraception – you know, the oral pill – and that was my biggest concern. I wanted to make sure I was on a tablet that didn't affect it. The computer randomly chooses the drug and I thought, I don't want to get a drug that isn't going to work for me.' (AD18)

Another (AD5) primarily wanted 'to get on with everyday life':

'As long as I can, this medication doesn't, just taking medication doesn't interfere with, I can still go out, can still have a few drinks, I said fine, I wasn't really too interested in the medical trial, as long as I'm staying fairly attack-free.' (AD5)

He was concerned that the trial 'might be a bit of a disruption' but also rejected taking part in clinical trials as frightening, just not 'my thing' and a 'last resort' only in the event of being unhappy with his treatment. The third (AD12) based his decision to decline on his desire to avoid the treatment a brother, also treated for epilepsy, had been prescribed. The brother's experience of that treatment had been negative, and AD12 realised he might be randomised to that treatment if participating in SANAD:

'The fact was I wasn't coming into this blind, my brother has been through all this, so I had a reference point, otherwise I would have gone in there thinking I had no idea. .... When we got to the bit about the clinical trial, once I had established that I might land, because it is random, on the one that my brother definitely had problems with, I said to be honest that is putting me off and I said I would rather do it on a one to one basis.' (AD12)

His desire not to participate outweighed the fact that, in principle, he supported the need for clinical trials and expressed willingness to act as 'a guinea pig'. The fourth (AD20) wished to take a proven clinical route, rather than, as he perceived it, accepting the 'unknown' route of participating in a clinical trial. Accordingly, there were elements of the trial he 'wasn't willing to go along with' particularly the risk of drug side effects:

'So I thought as I weighed them up, there were other sorts of side effects that could or could not arrive that I wasn't willing to go along with, so that's why I was turned off at that stage.' (AD20)

This individual was also reluctant to accept the diagnosis of epilepsy; and so to accept the need for treatment, either within the framework, or independent of a clinical trial.

One adult (AD14) expressed the desire not to be involved in SANAD based on his views about its purpose and his belief that 'whether I get better faster or not' was not a consideration:

'The big question mark is if there are two existing drugs which adequately treat seizures, then what is the reason for trying another four, except for perhaps reasons of economy or perhaps reasons of politics were other pharmaceutical companies are being let in to the scheme of things for purely economic or political reasons nothing to do with whether I get better faster or not. And you will just have to excuse me for being slightly cynical about these things. And I don't want to be involved in that.' (AW14)

So, despite initially agreeing to participate, he almost immediately withdrew agreement. Again, his desire not to participate for the reasons stated outweighed his altruistic view that trials were 'a positive activity to be involved in'. Hence, like those who agreed to participate, these five also acted in what they saw as their own best interests (see Figure 1).

### Aspects of SANAD that influenced patients' decision-making

#### Perception of treatments as tried and tested

As previously described, SANAD was a Phase IV pragmatic trial involving drugs already available through the NHS and about which a considerable amount of information about efficacy and side effects was already available from earlier phase trials. Unsurprisingly, this aspect of the trial was very important to informants who were concerned about their safety; and several expressed willingness to participate based on their understanding that the drugs were not really new and 'experimental', but already effectively 'tried and tested' and therefore safe:

'Well it was fine, because it wasn't like it was, they just plucked, like somebody had just discovered something and said well, you're trying that!' (PA2)

Indeed, they were able to be reassured on this point by the wording in the patient information leaflet, where it was stated that clinical trials had shown them to be safe, but that there was no good information as to whether they were 'more effective or safer' than the older drugs.

One parent, who had initially intended to decline participation because she did not want her child to have 'untested medicines', was persuaded by reassurances from the doctor of the extent of evidence of their safety:

'He [*consultant*] said I would like you to participate, and that's when I started asking questions – Are they safe? Have they been tested? How much do you know about them? And things like this and he said of course they are safe, they wouldn't have been passed, we know quite a lot about them, but what we don't know is whether they work any better than the old type of drugs. No, I'm not worried, I just think at the end of the day, doctors wouldn't prescribe something that is going to do my child any harm. I do have a lot of faith in the health system.' (PA19)

#### Perception of treatments as equivalent

Among informants in this study, willingness to participate in SANAD appeared to rest less on an appreciation of clinical equipoise (i.e. that there is no consensus about the comparative merits of the treatments being tested [38]) than on a perception of treatment equivalence (i.e. that the drugs under scrutiny were all broadly similar). A number of informants who consented referred – without being prompted by the researcher – to the similarity of the treatments involved:

'He said you have got five or six different tablets and they all do basically the same job, but you have got different side effects and then he showed me the booklet, I read it though and he said would you like to go on part of the study? And I said yes, straight away. I thought if they all do the same job, it's no bother to me, side effects or no side effects.' (AA10)

Based on their descriptions of the encounter with the recruiting clinician, these references to the equivalence of treatments seemed to have originated from the clinician's perhaps overly optimistic emphasis on the similarity of the various treatments, rather than the possibility of difference.

In contrast, for those declining to take part, the emphasis was not so much treatment equivalence as the uncertainties around SANAD. All four informants who declined from the outset and the one who initially consented and subsequently withdrew all expressed discomfort about what they perceived to be uncertainty about the process and outcome of participation:

'It is an unknown quantity, I don't want to be involved in something like that where the outcome is uncertain – the outcome must be uncertain, because otherwise why bother to do a study.' (AW14)

'It is difficult to make choices, you know what I mean, I have no idea of medical matters as I said so I really, rather than go down a path which was to my mind somewhat unknown I preferred the path that had already been proven.' (AD20)

Three of these individuals explicitly rejected randomisation and the idea that 'I might just land on any one' (AD12) of the drugs involved.

#### Perception of participation as voluntary and reversible

The perception that participation in the trial was voluntary and reversible was another important influence on informants' decision-making. Many informants emphasised the voluntariness of their engagement with SANAD, and that they were not put under pressure to do so. Though several informants declared themselves unbothered and even actively in favour of randomisation, many raised the reversibility of their initial decision as important, both of the decision to participate at all and of aspects of the trial such as the choice of drug. This element of the trial design appeared especially important to the parents of children who entered, 'because nobody wants to put their kids in danger, no matter what for' (PA4). Informants clearly perceived that they had the option to stop or adjust the drug treatment easily, that there was 'a get out clause' in the words of one parent (PA19), *despite *taking part in a trial:

'He [*consultant*] put me under no pressure whatsoever. He said if at any time you want to stop it then you can do, he said there is no pressure at all, you can stop it at any time and go back to the old drug.' (PA22)

'I was concerned about the tablets having [*side-effects*] and [*nurse specialist*] was saying that it's only rare and should it happen, the moment it happens, we will change you off that one and put you on a different one.' (AA15)

In summary, then, informants' perceptions of the nature of the SANAD trial were highly influential in their decisions to agree or decline to participate. Broadly speaking, informants either perceived the trial as potentially beneficial (or at least unlikely to be harmful) and focussed on participation as easily reversible – and so agreed to participate; or as potentially harmful (or at best unlikely to be beneficial) and focussed on the uncertainties involved – and so declined to participate.

## Discussion

In agreeing to participate in a clinical trial, patients should first accept clinical definitions of disease and the need for treatment, then be willing to entertain the possibility of clinical equipoise about which treatment to offer and the need for evidence to resolve it, the role of clinical trials in providing such evidence, and the need for randomisation as an essential element in trial design. All these elements were detectable across the range of comments made by informants who agreed to participate in SANAD. These individuals all accepted the clinician's definition of their symptoms as meaning they had epilepsy, and his/her recommendation that they therefore start treatment; understood that a number of treatment options were available; and appeared to accept that these were treatments about which some uncertainty persisted even though much was already known. Some, though not all, were explicit in their acceptance of randomisation; and most believed that they could reverse their decision easily and without implications for their condition or continuing treatment. Some also explicitly and others implicitly expressed their trust in the clinician, another element previously shown to be critical to participant agreement [23,39,40]. Importantly, several felt able to be reassured about the safety of the treatments under test and none expressly rejected any aspects of trial design. In contrast, the few informants who had declined to participate in SANAD accepted some but not all of these elements. One decliner was reluctant to accept the diagnosis of epilepsy; another accepted the diagnosis but was unconvinced about the need for treatment; two rejected the principle of randomisation, preferring treatment to be given 'on a one-to-one basis'. Like the trial non-participants in the study by Featherstone and colleagues [19] these individuals had clear and specific beliefs and treatment preferences, the latter potentially unattainable due to the process of randomisation; and all perceived that participation was potentially harmful.

The common element upon which both participants' and decliners' decisions hinged was thus their perception of whether participation would be beneficial or harmful. In Snowdon's study [41] of parents' decision-making about a neonatal treatment trial, those who accepted participation saw the research as either desirable, acceptable or not an issue, and the risks as worth taking; whereas for parents who declined participation, there was a high level of discomfort over the notion of experimentation and the risks were seen as unacceptable and clearly not in their child's best interests. Snowdon comments that, 'decliners placed it [*the study*] outside of the therapeutic setting in the unsettling realms of experimentation' and, not perceiving any particular benefits to participation, gave more prominence to the possible risks. A similar process seemed to be in train in SANAD despite the very different clinical setting – those agreeing to take part generally saw the risks of doing so as acceptable or not an issue, in this case because of the 'tried and tested' nature of the treatments and their broad similarity. In contrast, the five decliners saw taking part as 'an unknown quantity' and, like the decliners in Snowdon's study, defined the risks of being randomised to an unsuitable drug as unacceptably high and not in their best interests.

Two important reasons given for participation in SANAD were sympathy for the aims of the research and a sense of duty to take part. Informants spoke of the trial as a good idea, and as contributing to scientific knowledge; and themselves as wanting to be, in Harris's [42] words, 'public-spirited'. In this sense, our study mirrored findings of other earlier studies and supported previous claims that altruism is a key driver for participation. Their comments suggested that most would, if presented with it, acquiesce to Harris's claim that we all 'have an obligation in justice to contribute to the social practice which produces' medical advances. However, as described above, none of our informants cited only public-spirited reasons for participation – all also gave reasons related to self-interest. The term 'weak altruism' [43], though originally proposed as descriptive of a situation where patients consent to take enter a trial only because they perceive 'no positive net difference' between treatments and so do not expect to lose out, may usefully be extended to the describe the situation in SANAD where informants were happy to help others, but only where they could also help themselves. However, the nature of the SANAD trial also allowed informants to operate within the original definition of 'weak altruism', of being altruistic only 'in the weak sense that they are actively consenting to randomisation instead of passively accepting a default treatment' [43]. As previously described, SANAD was a trial of treatments already available outside the research context, the advisability of starting treatment had been discussed, and informants were often reassured by their discussions with the recruiting doctors that there were no net differences between treatments. These conditions may well have enabled them to be weakly altruistic, without any significant infringement to self-interest.

Corrigan [44] has noted a high level of ambivalence among the general public with regard to new or experimental drugs. The finding that informants who agreed to participate in SANAD were often clearly influenced by their understanding that even the 'new' drugs were not really new as they had been tested and were safe (and were in all likelihood 'better' than the older drugs) is therefore of some importance, in as much as it contributed to their being able to frame it as a low-risk enterprise. Previous studies [20,32] have highlighted that the issue of clinical equipoise plays an important role in patients' willingness to participate in clinical trials, those unwilling to accept the concept of equipoise being less likely to participate. In our study, however, it is possible that in some cases clinicians' explanations of the trial led to a situation where equipoise played a minor role in informants' decision-making compared to their perceptions of the *equivalence *of the treatments. Since we were unable to tape-record recruitment consultations, we are unable to clarify to what extent the emphasis on equivalence rather than equipoise reflected the clinicians' emphasis or informants' interpretations. However, this subtle difference in the way informants came to perceive the trial question seems to have been instrumental in their willingness to take part, suggesting further exploration of how these related but distinct aspects of treatment can be conveyed to patients is warranted. Both the focus on equivalence and the misinterpretation of the process of randomisation as nonetheless involving individualised care suggest some informants made their decisions about participation in SANAD based on a 'therapeutic misconception' about the risks involved. This, in turn, raises a question as to whether clinicians may have been sometimes overly optimistic or overly simplistic in their presentation of information about the various treatments or insufficiently clarified the impact of features of clinical trial design, and so exercised what has been described as 'unwitting coercion' [45] on potential participants. Our findings concur with those of Jenkins et al [46], that there may be areas of discussion that can be improved. The importance of identifying and addressing reasons for such misconceptions in future trials is self-evident.

The centrality of trust in relation to healthcare, and the increasing tendency towards mistrust have recently been highlighted by a number of authors [47,48]. In our study, as in others recently reported [39,40,44], trust in the doctor seemed to be an important element in the process of recruitment. Though it was an issue only infrequently raised explicitly, informants often made statements about their decision which were reflective of trust: for these patients, clinicians' reassurances about the safety and equivalence of treatments may have served as a signal to acceptance of randomisation. Our finding parallels that of Corrigan [44] who concludes that in the context of a clinical trial, where patients are seeking advice and reassurance about treatment from the doctor, 'a request to consent can be interpreted as guidance to consent.'

Harris [42] argues that at least where the costs and risks involved are minimal, people will want to participate in medical research. Overall, and in marked contrast to the situation in previously reported trials that compared irreversible treatment strategies [19,20,32]) for often life-threatening conditions [5,10,17-20], SANAD would seem to represent a low-cost, low-risk trial making few demands upon its participants beyond normal clinical care. However, the differentiation Harris makes between 'degree of danger' and 'probability of occurrence of danger' is highly relevant in the context of SANAD, since in relation to treatment of epilepsy the degree of danger is small (side effects of AEDs are generally of low severity whereas many people fear experiencing further seizures), even though the probability of occurrence is large (most people taking AEDs report side-effects [49]). Thus, the conditions of SANAD allowed the vast majority of patients approached to agree to participation, but led a few for whom the probability of occurrence of danger was too high to decline.

Our study was limited by its small sample size, the failure to interview all those initially agreeing to take part and the fact that none of the parents who took part had declined participation in SANAD. Informants were drawn from only four of the 90 centres that took part in SANAD and although we have no reason, based on analysis of the data collected for the main trial, to suppose that our 23 'participation study' informants were in any way different from others recruited (or not) to SANAD, we cannot be certain of this. The loss of 15 people from our original sample may reflect the timing of the recruitment process, since patients recruited to SANAD had just had a diagnosis of epilepsy and the advisability of treatment confirmed. It seems likely that for some, once they left the clinic and had time to reflect on events, taking part in research ceased to be a priority compared to the magnitude of the clinical considerations presented. Like Corrigan [44], we recognise that our informants may even have felt overwhelmed by the research process and that willingness to enrol in the participation study might have been enhanced had we been able to separate study recruitment from the clinical encounter. A further limitation previously highlighted was that we were unable to tape-record the SANAD recruitment consultations, so that our account of events rests solely on the recall and interpretations given to them by informants.

## Conclusion

Despite these limitations and the uncontroversial nature of SANAD, our data suggest that further research is needed to explore 'the myth and reality' [50] of trial recruitment processes and informed consent; and of patient motivations, interpretations and misinterpretations. In particular, future examination of the concept of 'altruism' demands a more nuanced approach; doctors' individual approaches to information giving about trials and the possibility of 'unwitting coercion' needs further consideration; as does the possibility of therapeutic misconception even in apparently straightforward trials. Our findings have practical and ethical implications both for feedback to recruiting clinicians in SANAD and for training of future trial recruiters.

## Declaration of competing interests

The author(s) declare that they have no competing interests.

## Authors' contributions

KC conducted the fieldwork and analysis. AJ conceived and designed the study, and participated in the analysis of the data. KC and AJ jointly drafted the manuscript. Both authors read and approved the final manuscript.

## References

[B1] Chard JA, Lilford RJ (1998). The use of equipoise in clinical trials. Soc Sci Med.

[B2] Prescott R, Counsell C, Gillespie W, Grant AM, Russell IT, Kiauka S, Colthart IR, Ross S, Shepherd SM, Russell D (1999). Factors that limit the quality, number and progress of randomised controlled trials. Health Technol Assess.

[B3] Ross S, Grant A, Counsell C, Gillespie W, Russell I, Prescott R (1999). Barriers to Participation in Randomised Controlled Trials: A Systematic Review. J Clin Epidemiol.

[B4] Robinson EJ, Kerr C, Stevens A, Lilford R, Braunholtz D, Edwards S (2004). Lay conceptions of the ethical and scientific justifications for random allocation in clinical trials. Soc Sci Med.

[B5] Fallowfield LJ, Jenkins V, Brennan C, Sawtell M, Moynihan C, Souhami RL (1998). Attitudes of patients to randomised clinical trials of cancer therapy. Eur J Cancer.

[B6] Slevin M, Mossman J, Bowling A, Leonard R, Steward W, Harper P, McIllmurray M, Thatcher N (1995). Volunteers or victims: patients' views of randomised cancer clinical trials. Br J Cancer.

[B7] Bevan EG, Chee LC, McGhee SM, McInnes GT (1993). Patients' attitudes to participation in clinical trials. Br J Pharmacol.

[B8] Larson E, McGuire DB (1990). Patient experiences with research in a tertiary care setting. Nurs Res.

[B9] Cassileth BR, Lusk EJ, Miller DS, Hurwitz S (1982). Attitudes toward clinical trials among patients and the public. JAMA.

[B10] Hudmon KS, Stoltzfus C, Chamberlain RM, Lorimor RJ, Steinbach G, Winn RJ (1996). Participants' Perceptions of a Phase I Colon Cancer Chemoprevention Trial. Control Clin Trials.

[B11] Mattson ME, Curb JD, McArdle R (1985). Participation in a clinical trial: the patients' point of view. Control Clin Trials.

[B12] Cunny KA, Miller HW (1994). Participation in clinical drug trials: Motivations and Barriers. Clin Ther.

[B13] Welton AJ, Vickers MR, Cooper JA, Meade TW, Marteau TM (1999). Is recruitment more difficult with a placebo arm in randomised controlled trials? A quasirandomised, interview based study. BMJ.

[B14] Searight HR, Miller CK (1996). Remembering and interpreting informed consent: a qualitative study of drug trial participants. J Am Board Fam Pract.

[B15] Sanchez S, Salazar G, Tijero M, Diaz S (2001). Informed consent procedures : responsibilities of researchers in developing countries. Bioethics.

[B16] Lawton J, Fox A, Fox C, Kinmonth AL (2003). Participating in the United Kingdom Prospective Diabetes Study (UKPDS): a qualitative study of patients' experiences. [see comment]. Br J Gen Pract.

[B17] Snowdon C, Garcia J, Elbourne D (1997). Making sense of randomisation: responses of parents of critically ill babies to random allocation of treatment in a clinical trial. Soc Sci Med.

[B18] Schutta KM, Burnett CB (2000). Factors that influence a patient's decision to participate in a phase I cancer trial. Oncol Nurs Forum.

[B19] Featherstone K, Donovan JL (1998). Random allocation or allocation at random? Patients' perspectives of participation in a randomised controlled trial. BMJ.

[B20] Mills N, Donovan JL, Smith M, Jacoby A, Neal DE, Hamdy FC (2003). Perceptions of equipoise are crucial to trial participation: a qualitative study of men in the ProtecT study. Control Clin Trials.

[B21] Mohanna K, Tunna K (1999). Withholding consent to participate in clinical trials: decisions of pregnant women. Br J Obstet Gynaecol.

[B22] Ellis PM (2000). Attitudes towards and participation in randomised controlled clinical trials in oncology: A review of the literature. Ann Oncol.

[B23] Gammelgaard A, Rossel P, Mortensen OS (2004). Patients' perceptions of informed consent in acute myocardial infarction research: a Danish study. Soc Sci Med.

[B24] Jenkins V, Fallowfield L (2000). Reasons for accepting or declining to participate in randomized clinical trials for cancer therapy. Br J Cancer.

[B25] Emanuel EJ, Patterson WB (1998). Ethics of randomized clinical trials. J Clin Oncol.

[B26] Hepworth J, Paine B, Miles H, Marley J, MacLennan A (2002). The willingness of women to participate in a long-term trial of hormone replacement therapy: a qualitative study using focus groups. Psychol Health Med.

[B27] Edwards SJL, Lilford RJ, Hewison J (1998). The ethics of randomised controlled trials from the perspectives of patients, the public, and healthcare professionals. BMJ.

[B28] Lidz CW, Appelbaum PS, Grisso T, Renaud M (2004). Therapeutic misconception and the appreciation of risks in clinical trials. Soc Sci Med.

[B29] Joffe S, Cook EF, Cleary PD, Clark JW, Weeks JC (2001). Quality of informed consent in cancer clinical trials; a cross-sectional survey. Lancet.

[B30] Schaeffer MH, Krantz DS, Wichman A, Masur H, Reed E, Vinicky JK (1996). The impact of disease severity on the informed consent process in clinical research. Am J Med.

[B31] Roberts F (2002). Qualitative differences among cancer trial explanations. Soc Sci Med.

[B32] Donovan J, Mills N, Smith M, Brindle L, Jacoby A, Peters T, Frankel S, Neal D, Hamdy F (2002). Improving design and conduct of randomised trials by embedding them in qualitative research: ProtecT (prostate testing for cancer and treatment) study. BMJ.

[B33] Marson AG, Tudur-Smith C, Gamble C, Shackley P, Williamson PR, Smith DF, Appleton R, Baker GA, Eaton B, Jacoby AJ, Chadwick DW Comparison of effectiveness and cost-effectiveness of new versus standard antiepileptic drugs The UK SANAD Study. Report to UK Health Technology Assessment Panel.

[B34] Reynolds EH (2002). ILAE/IBE/WHO Epilepsy Global Campaign History. Epilepsia.

[B35] Sander JWAS, Sillanpaa M, Engel J, Pedley TA (1997). Natural History and Prognosis. Epilepsy: a comprehensive textbook.

[B36] National Institute for Clinical Excellence (2004). Newer drugs for epilepsy in adults (Technology Appraisal Guidance 76) London.

[B37] Strauss A, Corbin J (1998). Basics of qualitative research: Techniques and procedures for developing grounded theory.

[B38] Freedman B (1987). Equipoise and the ethics of clinical research. New Eng J Med.

[B39] Zupancic JAF, Gillie P, Streiner DL, Watts JL, Schmidt B (1997). Determinants of parental authorization for involvement of newborn infants in clinical trials. Pediatrics.

[B40] Harth SS, Thong YH (1990). Sociodemographic and motivational characteristics of parents who volunteer their children for clinical research: a controlled study. BMJ.

[B41] Snowdon C (2005). Collaboration, participation and non-participation: decisions about involvement in randomised clinical trials for clinicians and parents in two neonatal trials. PhD thesis.

[B42] Harris J (2005). Scientific research is a moral duty. J Med Ethics.

[B43] Edwards SJL, Braunholtz D (2000). Can unequal be more fair? A response to Andrew Avins. J Med Ethics.

[B44] Corrigan O (2003). Empty Ethics: the problem with informed consent. Sociol Health Illn.

[B45] Little P (2002). Commentary: presenting unbiased information to patients can be difficult. BMJ.

[B46] Jenkins VA, Fallowfield LJ, Souhami A, Sawtell M (1999). How do doctors explain randomised controlled trials to their patients?. Eur J Cancer.

[B47] Ward P, Coates A (2006). We shed tears, but there is no one to wipe them up for us: narratives of (mis)trust in a materially deprived community. Health (London).

[B48] Scambler S (2002). Health and Social Change: a critical theory.

[B49] Baker GA, Jacoby A, Buck D, Stalgis C, Monnet D (1997). Quality of life of people with epilepsy: a European Study. Epilepsia.

[B50] Verheggen FWSM, van Wijmen FC (1996). Informed consent in clinical trials. Health Policy.

